# Postoperative pain after one-visit root-canal treatment on teeth with vital pulps: Comparison of three different obturation technique

**DOI:** 10.4317/medoral.17898

**Published:** 2012-02-09

**Authors:** Luis O. Alonso-Ezpeleta, Carmen Gasco-Garcia, Lizett Castellanos-Cosano, Jenifer Martín-González, Francsico J. López-Frías, Juan J. Segura-Egea

**Affiliations:** 1 Department of Endodontics, School of Dentistry, University of Zaragoza, Huesca, Spain; 2 Department of Pharmacology, School of Medicine, University of Madrid, Madrid, Spain; 3 Department of Endodontics, School of Dentistry, University of Sevilla, Sevilla, Spain

## Abstract

Objectives. To investigate and compare postoperative pain after one-visit root canal treatment (RCT) on teeth with vital pulps using three different obturation techniques. Study Design. Two hundred and four patients (105 men and 99 women) aged 12 to 77 years were randomly assigned into three treatments groups: cold lateral compaction of gutta-percha (LC), Thermafil technique (TT), and Backfill - Thermafil obturation technique (BT). Postoperative pain was recorded on a visual analogue scale (VAS) of 0 - 10 after 2 and 6 hours, and 1, 2, 3, 4, 5, 6 and 7 days. Data were statistically analyzed using multivariate logistic regression analysis. Results. In the total sample, 87% of patients experienced discomfort or pain in some moment between RCT and the seventh day. The discomfort experienced was weak, light, moderate and intense in 6%, 44%, 20% and 6% of the cases, respectively. Mean pain levels were 0.4 ± 0.4, 0.4 ± 0.3, and 1.4 ± 0.7 in LC, BT, and TT groups, respectively. Patients of TT group experienced a significantly higher mean pain level compared to other two groups (p < 0.0001). In TT group, all patients felt some level of pain at six hours after RCT. Conclusions. Postoperative pain was significantly associated with the obturation technique used during root canal treatment. Patients whose teeth were filled with Thermafil obturators (TT technique) showed significantly higher levels of discomfort than patients whose teeth were filled using any of the other two techniques.

** Key words:**Postoperative pain, root-canal obturation, root-canal treatment, Thermafil.

## Introduction

Pain is an unwanted yet unfortunately common sensation after root canal treatment (RCT) which commences a few hours or days after treatment and is always an unpleasant experience for both patients and clinicians (1-2). Root canal procedures are commonly believed to be the most painful dental treatment (3). The incidence of postoperative pain after RCT, mainly mild discomfort, was reported to range from 3% to 58% (4-5), but less than 12% of patients experienced severe pain (6).

The reasons for postoperative pain can be many including chemical, mechanical, or microbial injuries to the periapical tissues that result in acute inflammation (7). No significant difference in postoperative pain has been found when one-visit RCT was compared with two-visit treatment (2,8-11).

Mechanical factors, including over instrumentation or extrusion of root-filling materials, have been associated to the presence of postoperative pain (1,5), suggesting that root canal instrumentation and obturation techniques may influence postoperative pain. In fact, several studies have found correlation between the root canal instrumentation technique and postoperative pain (12,13). Nevertheless, no study has analyzed the influence of the obturation technique in postoperative pain.

The aim of this study was to evaluate and compare postoperative pain after one-visit RCT using three different obturation techniques.

## Material and Methods

Patient selection

The Ethics Committee of the University approved the investigation. Consecutive patients (n = 338) attending a trained endodontist (LOA-E) for primary RCT on only one tooth were invited to participate in this prospective study. All diagnoses were vital pulps, either asymptomatic irreversible pulpitis caused by carious exposures, either normal pulp of patient being referred for intentional endodontic therapy for prosthetic reasons. The individual diagnosis was confirmed by obtaining the dental history, periradicular radiographs, periodontal evaluation, percussion, and cold test (EndoIce; Coltène/Whaledent Inc, Cuyahoga Falls, OH). Previous NSAIDs or antibiotic treatment was recorded.

All patients were informed of the aims and design of the investigation, and the first 270 that agreed to participate and signed an informed consent were included in the study. Patients were supplied written instructions on how to assess and record the postoperative pain. However, only 204 patients (105 men and 99 women), with ages ranging 12 to 77 yr (mean: 42 ± 14 yr; median: 40) could be analysed finally because 66 subjects (dropout rate = 24%) did not completed and/or returned the questionnaires.

Selection of the obturation technique 

Ninety patients were randomly assigned to each one of the three obturation techniques: 1) treatment with cold lateral compaction of gutta-percha (Group LC); 2) treatment with Thermafil technique (group TT); and treatment with Backfill Thermafil obturation technique (group BT). After drop out, 80 patients were assigned to LC group, 61 to TT group, and 63 to BT group. ( Table 1) summarizes the distribution of tooth types and the obturation techniques.

**Table 1:**
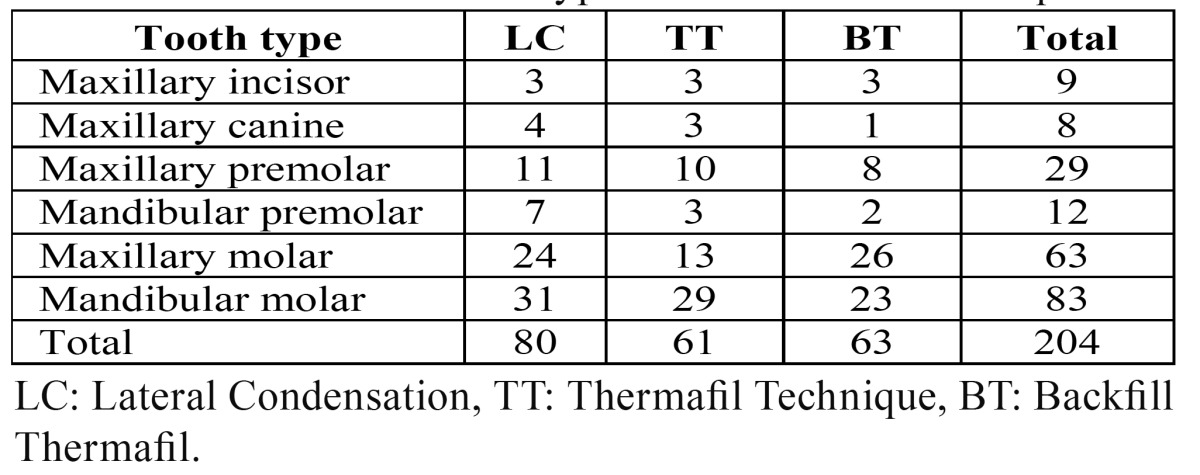
Distribution of tooth types and obturation techniques.

Endodontic protocol

All teeth were cleaned, shaped, and obturated during the patients’ first visit. Local anaesthesia was achieved by local infiltration with 4% articaine with 1:100,000 epinephrine (Laboratories Inibsa, Barcelona, Spain) or, in hypertensive patients (8%), 3% mepivacaine without vasoconstrictor (Laboratories Inibsa, Barcelona, Spain). After anesthesia, an endodontic access cavity was established by using 014 round carbide and Endo Z burs (Dentsply International, York, PA). Canals were prepared using the step-back technique with hand instrumentation. A glide path was established with stainless steel hand instruments up to a size #10. Cleaning and shaping preparation was achieved using middle-coronal preflaring carried out with Gates Glidden burs (sizes # 2, 3 and 4) (Dentsply Maillefer, Ballaigues, Switzerland). Patency was established and verified with #10 files. The ideal working length was determined using an electronic apex locator (Dentaport ZX, Morita, Tokyo, Japan) and periapical radiographs. The canal was cleaned and shaped by hand with K-Flexofiles (Dentsply Maillefer, Ballaigues, Switzerland). The final instrumentation size was determined as three sizes larger than the first file binding at the working length. Master apical files ranged from #25 to #50, depending on both root anatomy and initial diameter of the root canal. Apical preparation was completed using step-back at 1-mm increments. Irrigation was always performed with 5.25% NaOCl solution.

The teeth of the LC group were obturated with cold lateral compaction, using AH Plus (Dentsply De Trey GmBH, Germany) and gutta-percha (Aceone-Endo, Aceonedent. Co. Geonggi-Do, Korea). AH Plus was mixed according to the manufacturer’s instructions. The master gutta-percha cone was coated with AH Plus sealer, placed into the root canal, and fitted to the working length. Then, the gap for accessory cones was created using a #25 finger spreader (Dentsply Maillefer, Ballaigues, Switzerland). Excess gutta-percha was removed using a warm excavator.

Thermafil technique, with a plastic carrier, was used to obturate the teeth of the TT group. A thin layer of AH Plus sealer was placed into the root canal with a paper point. A Thermafil obturator (taper .04), selected after verification, was heated in the Thermaprep® Plus Oven (Densply, Maillefer, Ballaigues, Switzerland). The heated obturator was slowly inserted into the canal to the previously determined working length. A plugger was used to condense the coronal gutta-percha around the carrier until the gutta-percha hardened. Excess coronal gutta-percha and the plastic handle were removed with a round bur (ISO 016, Dentsply, Maillefer, Ballaigues, Switzerland) at 2000 rpm, without water cooling. Then, the gutta-percha was vertically condensed with pluggers nº 1/2 and 3/4 (Dentsply, Maillefer, Ballaigues, Switzerland).

The teeth of the BT group were obturated with the modified master cone heat-softened backfilling technique (Backfill – Thermafil, BT), as described by Da Silva et al. (14). A gutta-percha master cone (taper .02, Maillefer), coated with AH Plus sealer, was first introduced into the canal. The master cone was condensed with a #25 finger spreader (Dentsply Maillefer, Ballaigues, Switzerland) and a Thermafil point size 04/30 was used for back-filling of the canal. Excess coronal gutta-percha and the plastic handle were removed with a round bur and the root filling was vertically compacted as above.

In the three groups, the teeth were temporized using a sterile cotton pellet and Cavit (3M, St Paul, MN, USA).

Pain / discomfort assessment

Each patient received instruction on how to use a questionnaire for the numeric and verbal evaluation of pain / discomfort (5). The questionnaire contained a 10-cm visual analogue scale (VAS) (15) to assess discomfort / pain at 2 and 6 hours and 1, 2, 3, 4, 5, 6 and 7 days after the RCT was completed. The questionnaire should be completed and returned a week later, when they came to check up.

Statistical analysis

Raw data were entered into Excel (Microsoft Corporation, Redmond, WA, USA). The relationship between obturation techniques and clinical factors and postobturation pain was analyzed using odds ratio as well as logistic regression models based on bivariate and multivariate analysis (p < 0.05). Student t test was used to compare mean pain levels. The SPSS statistical software (version 11.0, SPSS Inc., Chicago, IL, USA) was used.

## Results

Seventeen percent of patients showed no postoperative pain, but 83% experienced discomfort or pain in some moment between the intervention and the seventh day. The discomfort experienced was weak, light, moderate and intense in 6%, 44%, 20% and 6% of the cases, respectively.

( Table 2) describes postoperative pain levels experienced by the participants when the LC obturation technique was used. Thirty per cent of patients experienced no pain at any time. The maximum postoperative pain level was “light”. The higher percentage of patients feeling pain was found at 6 hours (70%) and the first day (63%). The percentage of patients that felt pain decreased continuously from the six hours, being almost negligible on the seventh day after treatment (3%). The maximum pain intensity, at six hours, was 4, in 2.5% of the patients (n = 2), and the mean pain level was 0.4 ± 0.4.

**Table 2:**
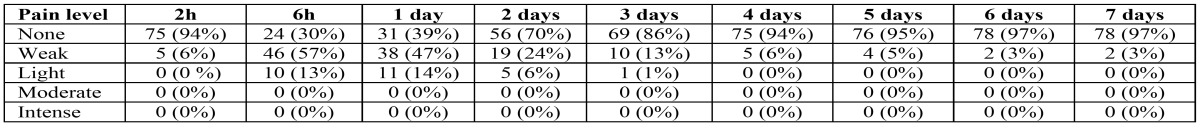
Postoperative pain experienced after root canal treatment (RCT) using lateral condensation obturation technique (LC). Patients (n = 80) completed a questionnaire containing a 10-cm visual analogue scale (VAS) (Huskisson 1974) to assess discomfort / pain at 2 and 6 hours and 1, 2, 3, 4, 5, 6 and 7 days after the RCT.

( Table 3) describes postoperative pain levels experienced by the participants when the BT obturation technique was used. Nine per cent of patients experienced no pain at any time. No patient felt neither moderate nor intense pain, and the maximum postoperative pain level was “light”. The higher percentage of patients feeling pain was found at 6 hours (91%) and the first day (76%). The maximum pain level in this group was 4, in 3% of the patient (n = 2), and decreased from the first day, and disappears on the fourth day. The mean pain level was 0.4 ± 0.3.

**Table 3:**
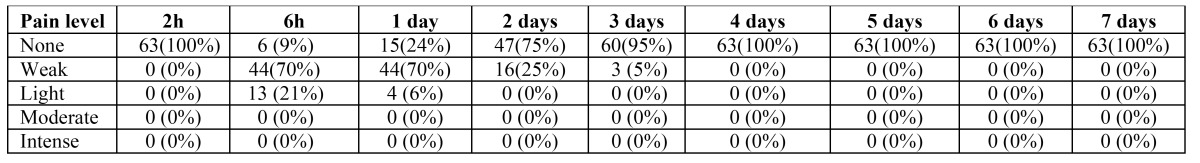
Postoperative pain experienced after root canal treatment (RCT) using Backfill Thermafil obturation technique (BT). Patients (n = 63) completed a questionnaire containing a 10-cm visual analogue scale (VAS) (Huskisson 1974) to assess discomfort / pain at 2 and 6 hours and 1, 2, 3, 4, 5, 6 and 7 days after the RCT.

Postoperative pain levels when the TT obturation technique was used are described in ( Table 4). All the patients felt some level of pain at six hours after the treatment. The maximum postoperative pain level was “intense”. Five percent of patients felt intense pain at six hours and 3% after one day. The higher percentage of patients feeling pain was found at 6 hours (100%) and after one day (97%). The percentage of patients that felt pain decreased continuously but slowly from the six hours to the seven day. At the 7 day, still 7% of patients showed some pain. The maximum pain level was 8 in 3% of the patient (n = 2). The mean pain level was 1.4 ± 0.7, significantly higher than that found in the groups LT and BT (p < 0.0001).

**Table 4:**
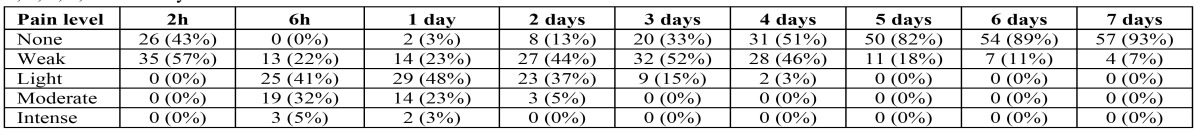
Postoperative pain experienced after root canal treatment (RCT) using Thermafil obturation technique (TT). Patients (n = 61) completed a questionnaire containing a 10-cm visual analogue scale (VAS) (Huskisson 1974) to assess discomfort / pain at 2 and 6 hours and 1, 2, 3, 4, 5, 6 and 7 days after the RCT.

**Figure 1:**
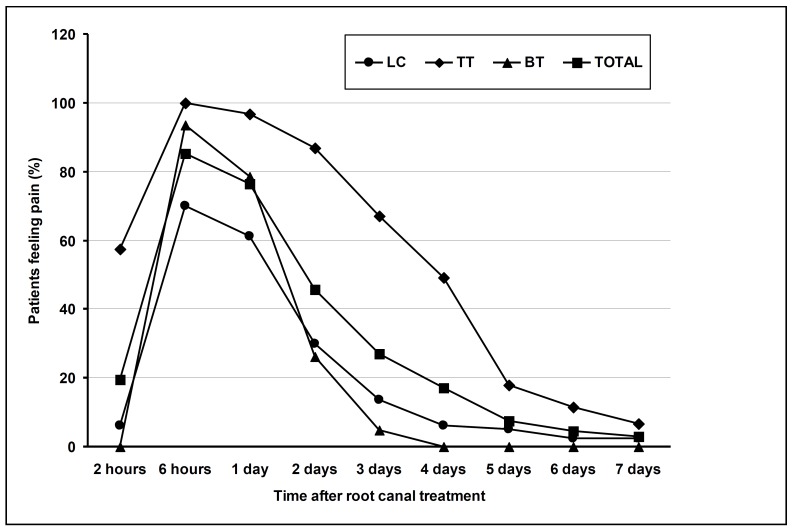
Percentage of patients feeling pain in the total sample and in each treatment group at different periods after root-canal treatment.

The percentages of patients feeling pain in the total sample and in each treatment group are shown in (Fig. 1). There were significant differences amongst the three techniques in relation to the pain (p < 0.01). At every time, the higher percentage of patients feeling pain corresponded to TT obturation technique (p < 0.01). The percentage of patient feeling postoperative pain in teeth obturated using LC and BT techniques decreased significantly after the first day, but in the teeth obturated with the TT technique maintained over fifty percent at 4th day and then decreased slowly.

**Figure 2:**
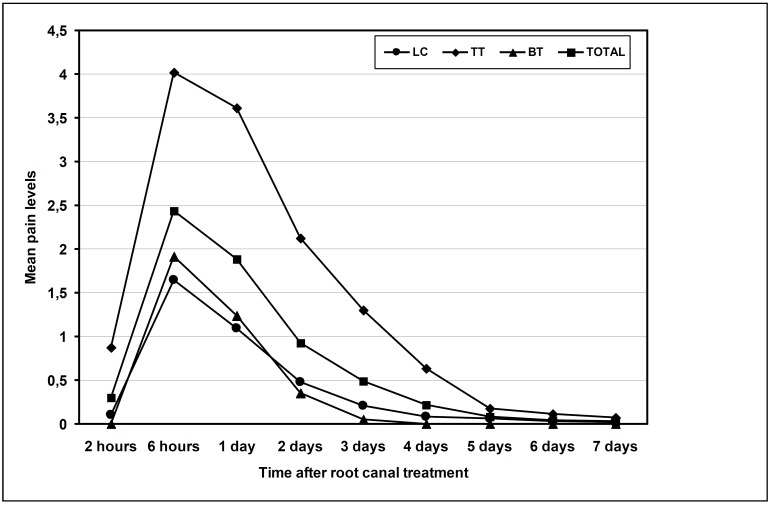
Mean pain levels in the total sample and in each treatment group at different periods after root canal treatment.

Mean pain level for all techniques was 0.71 ± 0.46 (Fig. 2). Mann-Whitney U test revealed a statistically significant difference between the median pain intensity depending on the obturation technique (p < 0.01). Higher mean pain level was reported at six hours after the treatment for all obturation techniques. At every time, the higher mean level of pain corresponded to TT obturation technique (p < 0.01).

Multivariate logistic regression analysis was run for the dependent variable “presence of pain after 6 hours”, adjusting for age, pulpal status, NSAIDs pre medication, obturation technique and treatment length as covariates. Analysis showed that age (p = 0.03) and pre medication with NSAIDs (p = 0.0021) were factors associated statistically to the presence of pain at 6 hours, but obturation technique did not correlated significantly (OR = 3.35; C.I. 95% 0.98 - 10.82; p = 0.053).

## Discussion

The purpose of this study was to compare the postoperative pain after endodontic therapy on teeth with vital pulps using three different obturation techniques. As long as we know, this is the first study analyzing this topic. The results of this study suggest that the TT obturation technique is significantly associated to higher postoperative pain levels.

Mild discomfort after root canal treatment is a common experience for patients (1). The reasons for postoperative pain, however, can be many (7). The main causes are mechanical, chemical, or microbial injuries to the periapical tissues that result in acute inflammation. In a clinical investigation, it is difficult to determine if a single or multiple factors elicit pain. If a root canal system was not cleaned properly, residual infection may cause exacerbation by imbalances in the host-bacteria relationship, synergistic or additive microbial interactions, or the presence of decisively pathogenic bacteria before the initiation of treatment (16). A mechanical reason may be overinstrumentation; chemical factors include the extrusion of medications, filling materials, or irrigants (5,17). In the present study, as only vital cases were included, persisting infection can be excluded as a cause of postoperative pain.

One of the main problems in studying pain is the patient’s subjective evaluation and its measurement. For this reason, the methodology used in assessing pain level is critical (18). In this study, as well as other studies on endodontic postoperative pain (19-20) a VAS has been used. In this study, pain has also been verbally quantified in order to a better understanding by patients.

Postoperative pain is common after endodontic treatment, so it is very important for the dentist to control this pain as well as to know how widespread the problem is (21). Root canal treatment must be carried out taking into account that instrumentation and obturation techniques can provoke periapical damage. Furthermore, several reports associate the extrusion of filling material to the presence of postoperative pain (1,5,17). In this study, cold lateral compaction technique (LC), obturation with Thermafil (TT) and a mixed obturation technique using Thermafil and a master gutta-percha cone (BT) (14) have been compared with regard to postoperative pain.

Results of the present study show that, although seventeen percent of patient showed no postoperative pain at any time, strikingly, 83% experienced some pain level during the week after the root canal treatment. This percentage of patient feeling pain is the highest reported in the literature (4-6). This result must be understood taking in mind that 1 and 2 pain levels (weak pain) are only “postoperative discomfort”. In addition, the Hawthorne effect, i.e., the change in the behavior of a subject because of the special attention and status received from participation in an investigation (22), can provoke that patients overestimate their pain levels. Considering no more than pain levels higher than 2, only 36% of patients reported pain. This result is in agreement with that of Gondim et al. (5), who compared post-endodontic pain using two irrigation techniques and reported 34% of pain after 4 hours. Ng et al. (6) reported a prevalence of postoperative pain after 24 hours of 40%. However, a review on endodontic postoperative pain reported a wide range of pain levels from 3% to 58% (4).

Analyzing the time course of the reported pain, results show that maximum pain level occurs six hours after the treatment in the three groups, as well as the percentages of patients feeling pain (85.3% in the total sample). This result agrees with the findings of other studies (2,23) that also registered the maximum postoperative pain level six hours after the treatment, when the anaesthetic effect has completely disappeared. At the first day, the total percentage of patients reporting some pain decreased lightly to 77%. Other studies have reported lower percentages of patients feeling pain after 1 day: Harrison et al. (1) found 38%, Koba et al. (24) reported 34%, and Ng et al. (6) observed 40%. This apparent discordance could be due, as previously has been stated, to the Hawthorne effect (22). Moreover, considering no more than pain levels higher than 2, only 36 % of patients reported pain after 1 day. Recently, Bagán et al. (25) have found that 55.5% of patients report having experienced pain after dental interventions, including conservative dentistry, oral surgery, endodontics, periodontics, and fixed prostheses. Endodontic interventions were associated with a greater severity of pain than conservative dentistry and fixed prosthesis, but mean pain levels were similar to those reported after oral surgery and periodontal procedures.

Results of the present study demonstrate that, at every time, mean pain level was higher in the patients treated with the TT technique. Moreover, mean pain level with TT technique was more than twice at all times, compared to LC and BT filling techniques. Strikingly, all the patients treated with the TT technique reported some pain at 6 hours (p < 0.01). Higher pain levels associated to TT obturation technique can be explained by the extrusion of gutta-percha that frequently occurs when this technique is used (26). Da Silva et al. (14) found overfilling in all teeth obturated with TT technique. However, Yesilsoy et al. (27) did not find correlation between sealer extrusion and post-obturation pain prevalence.

The type of root canal instrumentation technique can influence on the discomfort or pain experienced during endodontic therapy. Goreva and Petrikas (12) reported that “crown down” preparation using completely rotating profile instruments and GT rotary files proved to be effective as regards prevention of postoperative pain. Makeeva and Turkina (13) have analyzed the effects of the method of mechanical root canal treatment on emergence of pain after endodontic therapy. These authors compared sound tools of the Sonic system, ultrasound tools of the Satelec Suprasson system, full-wind tools of ProTaper and System GT as well as handy K-files, finding that the least risk of pain emergence after endodontic treatment occurs with tooth canal widening by crown-down technique.

In the present study, NSAIDs medication prior to root canal treatment was associated to higher levels of postoperative pain. Without doubt, the use of NSAIDs correlates with the presence of preoperative pain. Several studies have established that preoperative pain is a major determinant of postoperative pain or flare-up (6,28,29). Nevertheless, in other published studies, the presence and severity of preoperative pain did not appear to have any significant effect on the prevalence of post-obturation pain (10).

No correlation between pulpal status and post-operative pain levels has been found, in agreement with the result of Harrison et al. (1). However, it has been found that root canal treatment is more painful in teeth with irreversible pulpitis (29,30).

The outcome measure of future studies should be reported in terms of improvement or deterioration of pain level rather than mere prevalence of postoperative pain/flare-up (4).
